# Andean Tectonics and Mantle Dynamics as a Pervasive Influence on Amazonian Ecosystem

**DOI:** 10.1038/s41598-019-53465-y

**Published:** 2019-11-14

**Authors:** Tacio Cordeiro Bicudo, Victor Sacek, Renato Paes de Almeida, John M. Bates, Camila Cherem Ribas

**Affiliations:** 1 Instituto de Astronomia, Geofísica e Ciências Atmosféricas, Universidade de São Paulo, São Paulo, Brazil; 2 Instituto de Geociências, Universidade de São Paulo, São Paulo, Brazil; 3 Department of Zoology, The Field Museum, Chicago, USA; 4 Instituto Nacional de Pesquisas da Amazônia, Manaus, Brazil

**Keywords:** Biogeography, Geodynamics, Tectonics

## Abstract

The Amazonian landscape evolution is the result of the combined effect of Andean tectonism, climate and the Earth’s interior dynamics. To reconstruct the landscape evolution and its influence on paleoenvironmental variations within Amazonia since the Oligocene, we conducted numerical experiments that incorporate different surface and geodynamic processes, reproducing many paleogeographic features as inferred from the sedimentary record. We show that the evolution of the drainage pattern gradually reduced the area of sedimentation derived from the Guiana and Brazilian shields while expanded the Andean derived deposits during the Miocene, affecting the nutrient availability. First order biotic habitats were inferred from these paleogeographical reconstructions, showing an eastward expansion of várzea and terra firme forests and consequent retraction of igapó forests, with a millennial-scale reconfiguration of a mosaic of habitats in the lowlands. We conclude that this dynamism probably guided the observed patterns of speciation in the most biodiverse biome on Earth.

## Introduction

Understanding the drivers of biotic diversification in tropical regions, and particularly in Amazonia, is one of the great scientific challenges and requires interdisciplinary data acquisition and interpretation of biological, geological and paleoclimatic archives. From a biological perspective, divergent models have been proposed considering climatically-induced habitat fragmentation^1^, physically-induced population isolation^2^ or ongoing dispersal of populations across stable landscapes^3^ as the main drivers for speciation. These different views are based on current distributional patterns of organisms (mainly birds and primates) and more recently also on comparative genetic data overlain on selected paleogeographic scenarios for Amazonia during the Neogene. The scenarios are based on the interpretation of the geological and paleoclimatic record preserved in the Amazonian sedimentary basins. However, depositional and erosional gaps and age uncertainties hamper accurate paleogeographic reconstructions, particularly for the Miocene to the Early Pleistocene, and the spatially and temporally heterogeneous phylogeographic patterns that have been found so far^4,5^ are associated with a general timing that is more recent than traditional views on the assembly of Amazonian landscapes^6^, but older than several of the glacial periods, hampering most attempts of establishing clear relationships between Amazonian geologic and biotic histories. Therefore, the interpretation of growing molecular phylogenetic and phylogeographic databases is not currently matched with comparable paleogeographic information.

In order to create better constrained scenarios of the physical evolution of the Amazonian landscape at a scale of 10^7^ years, we present the results of numerical forward models considering the tectonic evolution of the Andean topography, surface processes, mantle dynamics and their subsequent effects on the spatial and temporal distribution of subsidence, uplift and sedimentation patterns in lowland Amazonia.

### Previous amazonian paleogeographic models

The record preserved for the sedimentary basins of Amazonia is far from complete and includes fossil-bearing fine-grained Miocene successions^7,8^, similar but poorly documented Pliocene units, and sandy Pleistocene units deposited on top of a major unconformity and characterized by a series of diachronous fluvial terraces^9–12^. Based on the interpretations of these sediments, the proposed depositional environments in Western Amazonia during the Miocene vary from a megawetland with marine influence^6^, to a set of lakes, swamps, floodplains and avulsive rivers^8^.

Additional sources of information that have been used to constrain the uncertainties regarding physical landscape evolution include data from the more complete sedimentary record on the Eastern continental margin^6,13–15^, which reveal that a transcontinental river brought sediment from the Andes to the Atlantic as early as the Middle Miocene, but even this date is discussed^7,8^. Most current views of a stable landscape in Amazonia in the last several millions of years are derived from a simplistic interpretation of such data as an indication of the establishment of the current drainage system.

Coupled numerical models for the crustal-lithospheric dynamics and Earth-surface processes^16^, on the other hand, indicate that the interplay between subsidence and sedimentation promote continuous dynamic changes in the landscape and rearrangements in drainage networks that are not restricted to the establishment of a transcontinental major river. Additionally, computational models for mantle convection^17–19^ showed that the subduction of the oceanic lithosphere under the western margin of South America could induce topographic perturbations of hundreds of meters in amplitude with a wavelength of thousands of kilometers. Nevertheless, these geodynamic models have not previously been used to develop scenarios useful to constrain the past distribution of biotic habitats. In order to achieve this, models must take into account all major factors that control topography and nutrient dispersal, including tectonically induced isostasy, flexure, erosion, sedimentation, and long wavelength topographic effects of mantle dynamics. Until now, these surface processes in Amazonia and the contribution of mantle convection have not been simulated concomitantly in the same numerical model.

### Coupled geodynamic scenarios and their controls: present numerical results

To simulate the interrelated evolution of surface processes and the Earth’s interior dynamics, we used a modified version of a numerical code^16^ that simulates the interaction of flexure of the lithosphere, Andean orogeny and surface processes of erosion and sedimentation. In the present version, we incorporate the influence of dynamic topography due to mantle convection derived from previously published maps of dynamic topography for northern South America^19^.

The combination of flexure of the lithosphere and dynamic topography results in the superposition of topographic perturbations of different wavelengths: while the flexural effects are more significant close to the Andes due to the topographic load of the Andean Cordillera, generating foreland sedimentary basins hundreds of kilometers wide and a few kilometers deep, the influence of dynamic topography can affect the entire continent, with amplitude of tens to hundreds of meters. We show here that the combination of these two processes results in a drainage dynamic that differs from the predictions of the contribution of these effects individually.

**Figure 1:**
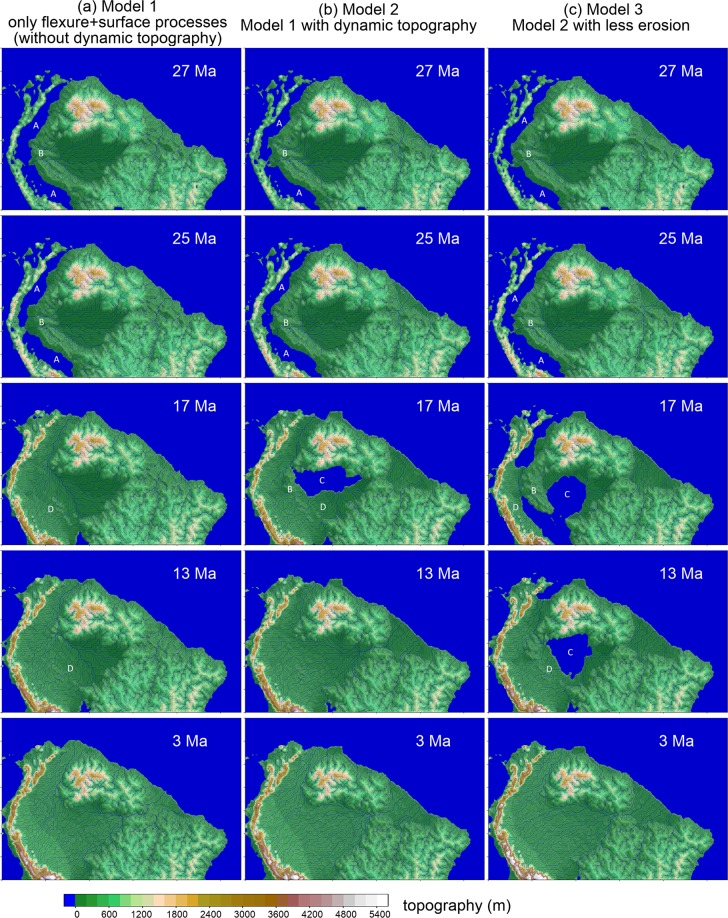
Landscape evolution for three numerical scenarios. The dimensions of the domain are 5450 km x 3500 km. (**a**) The first column represents the evolution of a model without the influence of dynamic topography, Model 1. The second and third columns present two models with the influence of dynamic topography, Models 2 (**b**) and 3 (**c**). Movies for the landscape evolution for these models are in the Supplementary Material (Movies S1–S3). The difference between these last two models is the erosion length scale of the basement *L*_*basement*_, a parameter that controls the erodibility of the basement rocks, and consequently, the rates of denudation of the Andean Chain (See Methods). In Models 1 and 2 the *L*_*basement*_ is equal to 600 km. In Model 3 the *L*_*basement*_ is equal to 800 km, which means harder basement rocks and less erosion rates when compared with Models 1 and 2. The white capital letters indicate the regions of the ubiquitous features cited in the text: A – Flexural foredeep; B – Flexural forebulge; C – Interior wetland or shallow lake; D – Andean sedimentary wedge. See text for details.

As a control scenario, we performed the simulation of the tectono-sedimentary evolution of the northern portion of South America without the influence of dynamic topography, analyzing only the interaction of surface processes, orogeny and flexure of the lithosphere (Model 1 - Fig. 1a). In this scenario, the crustal thickening in the Andes induces the flexural subsidence of the western margin of South America, creating a depression parallel to the cordillera, mainly below sea level, which corresponds to the foredeep depozone (Fig. 1a, images at 27 and 25 Ma). Depending on the chosen rate of crustal thickening in the Andes and the efficiency of the surface processes dynamics, the large domain covered by water may be connected with the ocean or may represent a deep interior lake. The foredeep depozone is separated from the interior of the continent by the forebulge, a topographic high created by the flexural effect of the load of the Andes over the continental lithosphere. This topographic high is a local water divide, preventing the Andean sediments from reaching the continental interior.

As the rate of the Andean erosion increases with time, the foredeep is continuously filled with sediments mainly derived from the cordillera. Finally, the foredeep depression is completely filled and the forebulge is covered by sediments, allowing the rivers to transport sediments from the Andes to the interior of the continent (Fig. 1a, images at 17 and 13 Ma). Until this time, the Equatorial Margin is not fluvially connected with Western Amazonia, and two independent drainage systems existed: (i) A small drainage system flowing towards the Equatorial Atlantic with headwaters on the eastern part of the Guiana and Brazilian shields; (ii) a drainage system in Western Amazonia with rivers originating on the Shields and the Andes meeting in a trunk river that flowed northward to the Caribbean Sea. With the progressive input of siliciclastic sediments derived from the Andes reaching the interior of the continent, Western Amazonia becomes connected with the Equatorial Margin, forming the transcontinental Amazon River (Fig. 1a, at 3 Ma). Depending on the efficiency of the surface processes and the initial topography assumed in the simulation, the timing for the connection between the two drainage systems varies by a few million years. It is important to highlight that the transcontinental connection observed in the numerical models is not a breakthrough event. Instead, it is part of a continuous process of landscape change.

Landscape evolution in the models under the influence of dynamic topography (Models 2 and 3, Fig. 1b,c, respectively) initially agrees with Model 1 for ages older than 20 Ma, reproducing a depression parallel to the Andean Cordillera along the foreland basins. However, after this first stage, the regional subsidence promoted by the subducting plate under the western margin of South America induces the creation of a shallow submerged area that extends more than one thousand km eastward from the flexural forebulge (Fig. 1b,c, images at 17 Ma). Depending on the magnitude of the dynamic topography and the erosion rate in the Andes, the forebulge can become partially isolated from the other continental areas, surrounded by aquatic environments for a few million years (Fig. 1c at 17 Ma). Increasing sedimentation in the foreland basin system combined with the change in dynamic topography leads to the progressive displacement of the submerged areas eastward (Figs. 1b,c), culminating in the joining of the Western and Eastern Amazonian drainage systems. After the drainage connection establishment, the large aquatic environment shrinks and the current Amazonian fluvial environments begin to form.

The numerical scenarios presented here are compatible with first order geological constraints: (1) paleoaltitude of the Andean orogeny (see Supplementary Information); (2) thickness of the sedimentary layer in the foredeep basins deposited during the simulated period^20^; and (3) the age of the onset of the Amazon River^13,15^. (Details about the comparisons are shown in the Supplementary Information).

Some of the main geographic features are ubiquitous in all model runs, whereas others are strongly dependent on timing and magnitude of particular geodynamic processes. The ubiquitous features are (see Fig. 1):

A. A flexural foredeep, adjacent to the cordillera, where a deep lake or marine gulf persists during the initial stages of main tectonic uplift in the Andes. This foredeep has its width controlled by the flexural rigidity of the lithosphere, ranging between 300 to 500 km, with aquatic environment enduring for nearly 13 million years. Based on published scenarios for the Andean uplift, the underfilled conditions in the foredeep probably lasted from Late Oligocene to Late Miocene^20^.
B. An uplifted range to the east of the foredeep, caused by the flexural uplift of the forebulge. The timing and amplitude of that range are correlated to the subsidence in the foredeep, and the modeled elevations are in the range of 100 to 200 m. In all simulated scenarios, the presence of the forebulge ridge guides the drainage of central Amazonia towards the North, to the equivalent modern Orinoco basin until the advance of the Andean-derived sediment wedge reaches and fills central Amazonia.
C. A thousands-of-kilometers wide depression, eastward of the flexural forebulge, caused by mantle dynamics (only in Models 2 and 3) that gave rise to either a shallow lake or active alluvial environments. The depression is established in central Amazonia during the foredeep-forebulge stage and persists afterwards, contributing to the reduction of the forebulge relief and finally connecting the eastern and western depression in a single Amazonian plain.
D. An Andean-derived sediment wedge advancing eastward, initially as deltas that grows slowly onto the foredeep (feature A) and later advances faster onto the shallower depression (feature C). In scenarios where the wide depression formed by mantle-dynamics is not a lake but alluvial plains, eastern and western drainage systems meet in a northward flowing trunk river.

### Historical distribution of habitats

**Figure 2:**
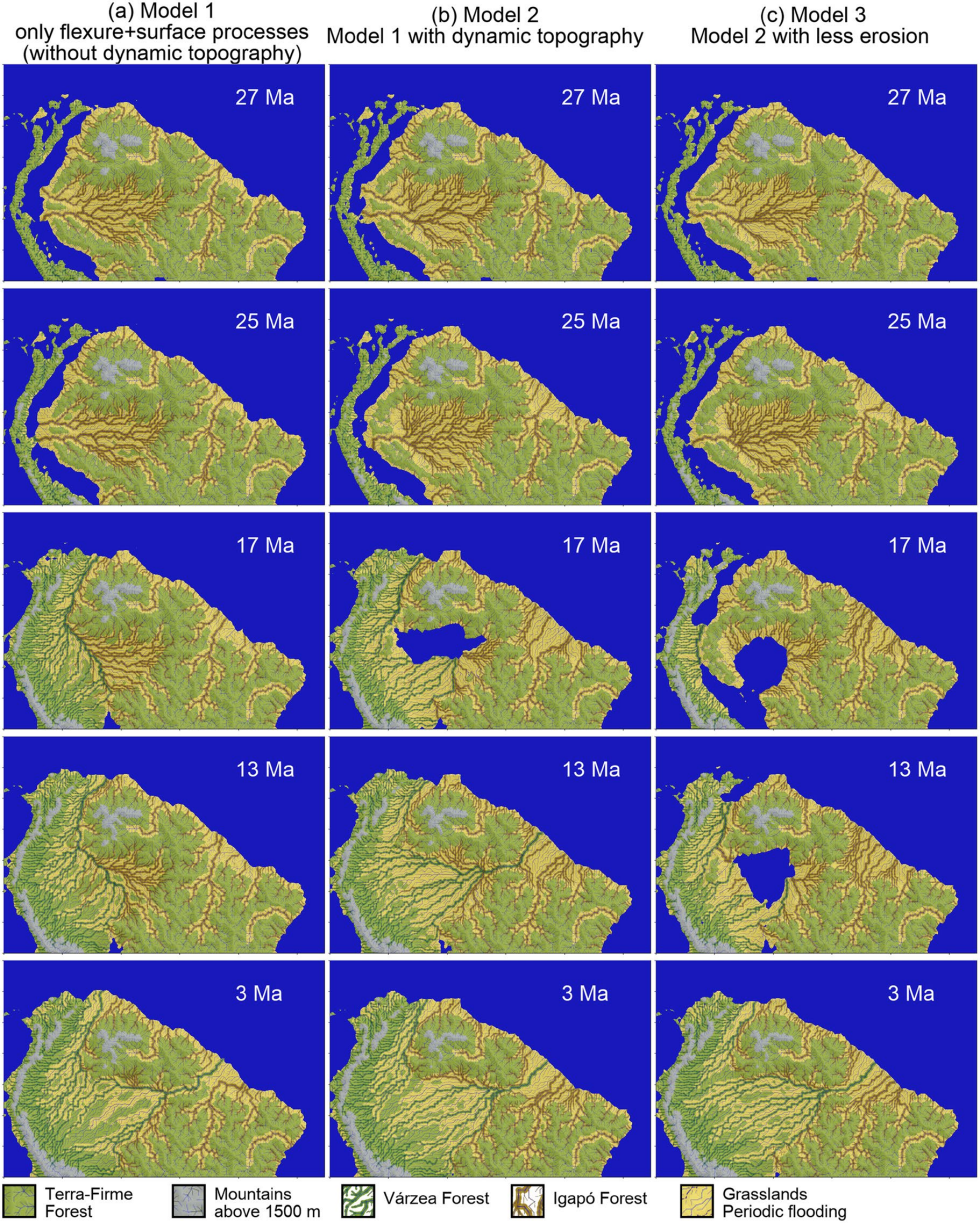
First order biotic habitats inferred from the paleogeographic scenarios shown in Fig. 1. The dimensions of the domain are 5450 km x 3500 km. (**a**) Model 1, (**b**) Model 2 and (**c**) Model 3. Movies for the habitats evolution for these models are in the Supplementary Material (Movies S4–S6). The procedure used to make this conversion is explained in “Methods”. The legend shows the interpreted habitats and details are described in the main text.

The paleogeographic scenarios achieved in our simulations can be translated into first-order biotic habitats (Fig. 2), considering the influence of flooding regime, permanent water bodies and availability of nutrients due to sediment sources. The premises for this interpretation, derived from the observation of modern environments and habitats, are:

1. Areas uplifted to a few hundred meters are protected from flooding and not high enough to prevent forest development, thus, they bear habitats analogous to the modern non-flooded forests (terra firme forest).
2. Areas undergoing subsidence and only partially filled by sediments develop permanent aquatic environments. Here, the interplay of subsidence and sedimentation results in a few meters deep water bodies that are not connected to the ocean, these regions may be subject to periodic exposure, bearing a mosaic of lakes, wetlands and floodplains, as opposed to simple huge shallow lakes.
3. Areas undergoing subsidence and filled by alluvial sediments are characterized by a mosaic of periodically flooded forests and open vegetation environments.
4. Sediments derived from the Andes are richer in nutrients than those derived from shield areas. As a consequence, flooded habitats on the floodplains of Andean-sourced rivers (várzea) differ markedly from those of shield-derived rivers (igapó).
5. Areas on the alluvial plain far from river flood waters but subject to periodic flooding, mostly by rainfall, develop open-vegetation, mostly shrubs and grasslands (white sand vegetation and savanas).
6. Flooded forests might be replaced by terra firme forest regionally due to river base-level drop, caused either by changes in the water discharge to sediment flux relationship or to the retarded far-field effects of sea-level oscillation^21^.

Applying these criteria to interpret the numerical model provides some key conclusions regarding the large-scale evolution of the Amazonian habitats.

Large aquatic environments dominant in central Amazonia are gradually replaced by flooded habitats and grasslands. Várzea flooded habitats began as small areas on the eastern Andean slope and gradually expanded to the east as deltas prograde onto the initially predominantly aquatic environments. High-frequency (millennial-scale) shifts in the position of individual channels led to fast reconfigurations of a mosaic of flooded forest, floodplain grasslands and lakes in the numerical scenarios. Igapó flooded habitats developed adjacent to the shields and expanded at a slower pace due to the lower sediment yields. Eastern igapó and western várzea were initially separated by a large lake, and later by a northward flowing river that shifted its position to the east until the Andean-derived sediment wedge reaches the eastern divide and connects to the Atlantic drainage system.

Terra firme forests already existed on the shields before Andean uplift. At the early stages of Andean tectonics, two other areas of isolated or partially isolated terra firme forest were created, one on the lower altitude rims of the Andes and another on the forebulge ridge. In the numerical scenarios, the latter lasted for 10 to 14 million years and was then replaced by subsiding environments. The present-day occurrence of terra firme forest on top of Late Pleistocene alluvial sediments in central Amazonia^9–11,22^ is an evidence of shift from alluvial environment bearing flooded forests to never flooded habitats due to an episode of base-level fall^12^. This implies cycles of expansion and retraction of these habitats in response to base-level oscillation.

The models suggest that the ongoing sedimentation primarily from the Andes continues to put significant and formative pressure on the movement of water across central Amazonia up to the present.

### Amazonian biota diversification

These general patterns of expansion, retraction and connectivity of the main habitats of Amazonia during Andean orogeny must be taken into account for a more realistic interpretation of evolutionary processes that have influenced the origin and current distribution of Amazonian biodiversity. Instead of alternative “old” or “young” scenarios for the development of current landscapes, the models presented here argue for a continuously dynamic landscape dominated for long periods by distinct bodies of shallow water and associated habitats across western and central Amazonia. This dynamic history has probably had a strong influence on biotic evolution on multiple levels. The landscape history modeled here indicates that the modern river system, while significant in terms of reducing gene flow and shaping current species distributions, is a recent and probably ephemeral actor in a long evolutionary play.

Establishing correlated evolutionary responses of the biota is a logical next step in looking at these models. Here we discuss basic aspects from the perspective of the growing body of research on the diversification of Amazonian biota. Our goal is to highlight overall patterns in data with specific examples rather than to present an exhaustive review of all data sets.

**Figure 3:**
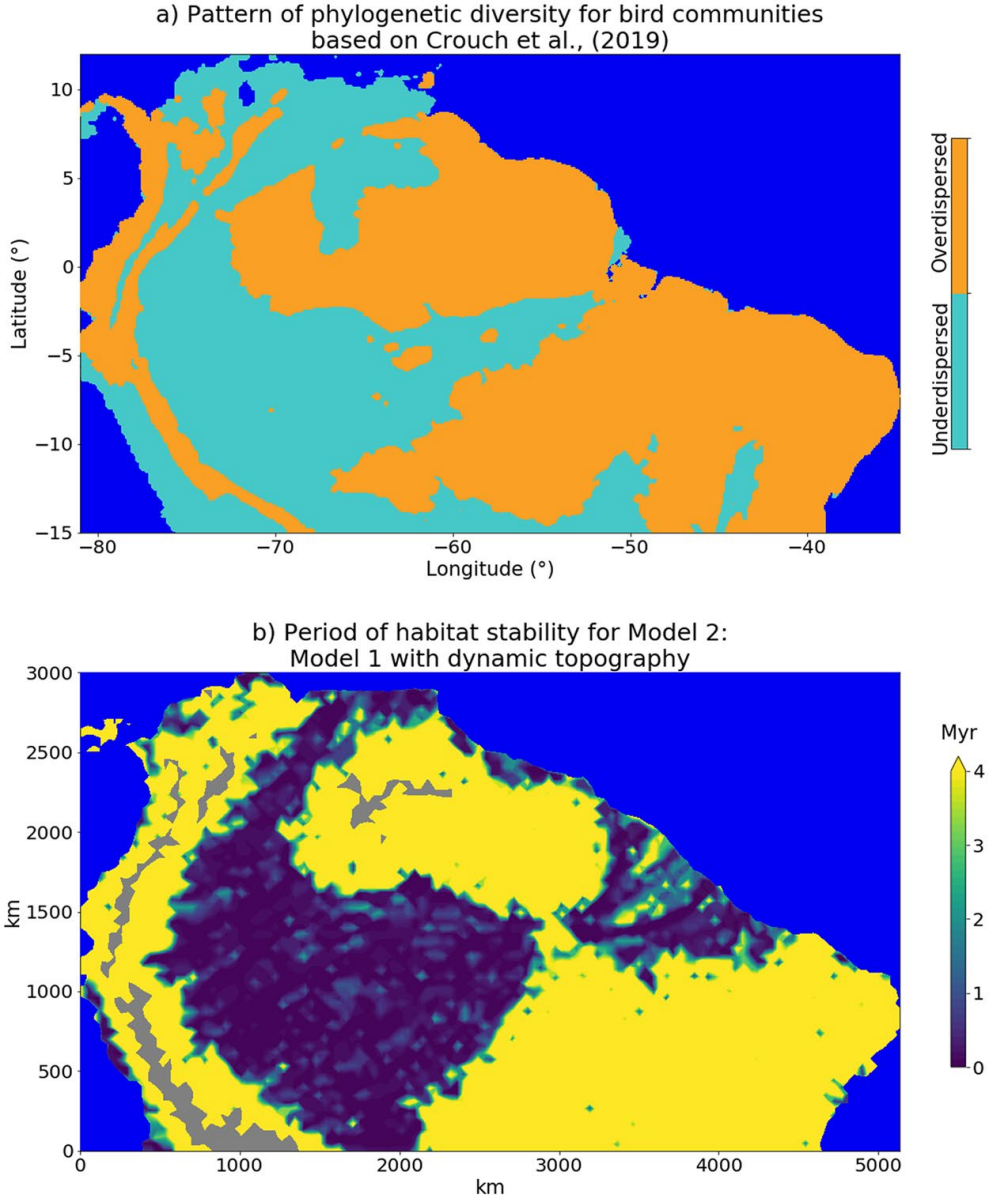
(**a**) Phylogenetic diversity of bird communities in northern South America (adapted from Crouch *et al*.^23^). (**b**) Period of uninterrupted habitat stability at the end of the simulation for Model 2 (the model with the dynamic topography contribution). The grey areas are regions higher than 1500 m. See Supplementary Information for equivalent maps for Models 1 and 3.

The literature about how current Amazonian communities are shaped is marked by the debate about the relative contributions of historical processes (biogeographical and climatic)^23^ versus differential dispersal^24,25^ not directly related to landscape features or its evolution. If dispersal is the main driver of current patterns of diversity, the phylogenetic diversity of current communities should have no clear spatial relationship with landscape history^25^. However, the results of the models presented here show that stable or unstable regions along the history of Amazonian landscape largely coincide with areas that have different patterns of passerine community phylogenetic structure^23^ (Fig. 3). A comparable result, but including fewer Amazonian localities was found for trees^24^ and snakes^26^, strongly suggesting a non-random relationship between Earth history, community assembly and diversity distribution through time.

Species diversity maps for more than 1700 passerine birds across northern South America show that present-day rivers do clearly influence species distributions across Amazonia^23^. However, based on patterns of phylogenetic diversity and species richness, Crouch *et al*.^23^ classified the passerine communities into “overdispersed”, representing regions with comparatively older and less related lineages, and “underdispersed”, with younger and more closely related lineages (see Fig. 3a). They conclude that geographical distribution of overdispersed and underdispersed regions is not associated with modern Amazonian rivers: Overdispersed regions lie mainly on the Guiana and Brazilian Shields and on the base of the Andes, while underdispersed regions are on the complement, the lowlands in Western and Central Amazonia. These two observed patterns in bird communities clearly correspond spatially to regions that sustained either an uninterrupted period of habitat stability or marked habitat change according to the models described here (Fig. 3b).

Overdispersed areas match regions of long-term habitat stability that harbor a mix of old and young lineages (yellow regions of Fig. 3b), while underdispersed regions correspond to areas where habitats continuously changed over the last 4 Ma (dark blue to light green regions of Fig. 3b), and communities were assembled mostly by recent speciation^5,23^. This agreement between interpretations derived from the numerical models and the broad analysis of passerine communities distribution^23^ strongly suggests for the first time a mechanistic and spatially explicit link between landscape evolution and biotic diversification in Amazonia. It is important to highlight that there is no specific event of speciation that might be correlated to particular events of landscape change, but rather high frequency dynamic changes in the habitats that are in agreement with apparently contradicting histories of vicariant barriers for different lineages^5^.

Most detailed phylogeographic studies published so far deal with a time frame that spans only the last stages of the model (less than 5 Ma), and completely sampled family level phylogenies are rare due to the intrinsic difficulties associated to resolving the taxonomy and sampling of Amazonian taxa. Even considering this temporal restriction, the models presented here help contextualize published phylogenies of Amazonian birds specialized in upland forests^2,5^. A recent comparative analysis of a large set of understory terra firme birds suggests that old lineages occur on the shields, with oldest splits happening between the Guiana shield and western Amazonia, and recent diversification centered in western and central Amazonia^5^. Other groups of Amazonian birds and primates have a diversification history associated with northwestern South America^27–29^, with ancestral lineages’ distributions optimized at the terra firme regions at the base of the Andes. The connection between Amazonas and Orinoco drainage systems has long been investigated also for the aquatic fauna, with clear evidence of past connection and dynamic river capture events up to the present^30–32^. Other clades of terra firme forest birds and primates, on the other hand, seem to have diversified in Amazonia from the southwestern forests at the base of the Andes, occupying the Amazonian upland forests when they became available in central Amazonia after 3 Ma^33–36^.

Recent studies of birds and primates specialized in seasonally flooded forests show a consistent pattern of past isolation between western and eastern lineages, with evidence of recent contact in central Amazonia^37^ or expansion from west to east^38^. Fish species diversity along the Amazon basin corroborates the long-time persistence of aquatic environments in the west, with high species richness, endemism, and similarity in species composition among western sub-basins^31,39^. Interestingly, Oberdorff *et al*.^39^ also report high endemism in the Tapajós and Xingu sub-basins, both located in the Brazilian shield, recovered as stable in the models presented here (Fig. 3b). Recent re-configuration of river basins is also supported, as fish species composition does not conform to the current delimitation of the main river basins^31^ and the lower richness of eastern fish communities indicates that the West-East colonization is still in progress, which is not compatible with a drainage system that has been stable for million of years^39^.

Thus, the hypothesis that western Amazonia was for a long period dominated by aquatic and flooded environments is in agreement with population genomic estimates for birds and primates, as well as with fish species composition and richness in Amazonian sub-basins. The dynamic nature of environmental evolution for Amazonia suggests that different taxa would potentially have very different histories superimposed in the same geographic scenario (Amazon basin) depending on their habitat affinities and on where the ancestors of extant lineages were distributed at specific periods of time. This explains the partial congruence of diversification patterns found so far and argues for more detailed studies taking nuanced landscape history and habitat specificity into account.

Through the years, researchers have suggested that Amazonia was a “museum” and the Andes a “cradle” where young taxa were evolving^40,41^. The geodynamic models presented here demonstrate that Andean uplift, which did lead to newly habitable areas at higher elevations, also directly contributed to a long history of dynamism and habitat heterogeneity in western and central Amazonia. These models contribute to solving a longstanding problem in the interpretation of the historical assembly of Amazonian biota, which had an old trigger (Andean uplift) but comparatively young speciation^2,5,42^, and agree with previous interpretations of the geological record that indicate a gradual response of the Amazonian landscape to Andean orogenesis^8^. The long debate on general instability versus stability in Amazonia as a whole seems overrated, and it is now important to test for associations between Earth history and biological diversification in a smaller and more specific scale, considering current habitat heterogeneity in the basin based on acceptance of a long dynamic history.

## Methods

The present tectono-sedimentary numerical model uses the same code presented by Sacek^16^ with two important changes: (i) here, for simplicity, the climate was simulated by a constant precipitation *V*_*R*_ rather than the orographic precipitation (see Table 1 for the model parameters); (ii) we included the contribution of dynamic topography derived from published geodynamic convection models^19^. Details about the equations for each process of the original numerical model can be found in the work of Sacek^16^.

**Table 1:** Fixed parameters in all simulated scenarios.

	Parameter	Description	Value
*Orogeny*	*k*_*or*_	Ratio between crustal thickening and actual relief of the Andes	0.24 Myear^−1^
*Flexure and Isostasy*	*T*_*e*_	Elastic thickness of the lithosphere	Onshore: 70 km
			Offshore + W. Andes: 15 km
	*ρ*_m_	Density of the mantle rocks	3300 kg/m^3^
	*ρ*_*c*_	Density of the crust and the sediment	2700 kg/m^3^
	*ρ*_*w*_	Density of the water	1030 kg/m^3^
	*E*	Young’s Modulus	1.0 × 10^11^ N/m^2^
	*ν*	Poisson Ratio	0.25
*Surface Processes and Climate*	*k*_*m*_	Diffusivity of marine sediment transport	1000 m^2^/year
	*L*_*shields*_	Erosion length scale of the shields: parameter related with the erodibility of the Shields rocks.	4000 km
	*L*_*basement*_	Erosion length scale of the basement:	600 km (Model 1 and 2) 800 km (Model 3)
	*L*_*sediments*_	Erosion length scale of the sediments	200 m
	*V*_*R*_	Effective precipitation rate	2.0 m/year
	*k*_*f*_	Erosional coefficient due to fluvial processes	0.08

### Setup of initial scenarios

The surface was constructed over an irregular mesh with 126 × 81 points, in which each point is associated with a Voronoi Cell. The georeferencing of this mesh was done by assuming that the bottom left and the top right corners of the mesh correspond, respectively, to the geographical coordinates 81°W, 16°S and 30°W, 15°N.

**Figure 4:**
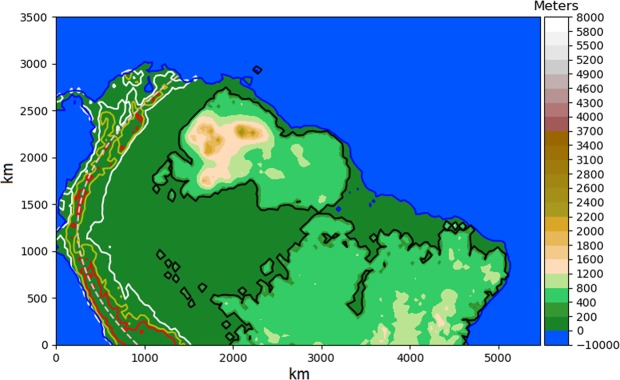
Initial topography and configuration of the numerical simulations. The colors indicate the topography. The black solid line delimits the region that corresponds to the shields, where the rocks are more resistant to erosion. The white, yellow and red contour lines mark the regions where the crustal thickening rate is 50 m/Myr, 350 m/Myr and 700 m/Myr, respectively. The pink dashed line along the Andes separates the limit between a low value of lithospheric elastic thickness, to the west of the curve and ocean, and high value, for the land areas.

We used the global relief model ETOPO1^43^ to create the initial topography for the simulations, which incorporates the major geomorphological features of northern South America at 30 Ma. Previous paleogeographic reconstructions^6–8^ allowed us to infer the main paleodrainage pattern and the geomorphological aspects in Late Paleogene. Therefore, from the ETOPO1 we preserved the high altitudes of the cratonic areas in the onset of the simulation. In the beginning, the altitude of the Andean Cordillera and the lowlands areas in Amazonia were set to 20 meters high. We added more 200 meters to the altitude of cratonic areas to account for the erosion and the dynamic subsidence of these regions during the simulated period. Additionally, to ensure a westward flow direction of the main rivers in Central and Western Amazonia at the beginning of the simulation, in accordance with previous paleogeographic reconstructions^6–8^, we added a topographic tilt dipping to the west and with a constant gradient of 1 meter per 60 km. The initial scenario constructed for our simulations is represented in Fig. 4.

### Incorporation of geodynamic processes

In our model, the orogeny and the dynamic topography are imposed in the numerical scenarios and are not affected by the surface processes and the flexural isostasy of the lithosphere. To simulate the orogeny in the Andean region, we imposed a crustal thickening rate *U* = *U(x, y)* proportional to the present topography of the Andean Cordillera *h*_*Andes*_, therefore, *U* = *k*_*or*_. *h*_*Andes*_, where the parameter *k*_*or*_ is the proportionality ratio between the crustal thickening ratio used in the simulations and the present Andean topography (see Table 1).

The dynamic topography maps used in the simulations were extracted from the website https://www.earthbyte.org/influence-of-subduction-history-on-south-american-topography. We used the contribution for Case 3, which is the scenario that incorporates more elements to the geodynamic modeling^19^.

The isostasy and flexure of the lithosphere compensate the loads over the surface. The vertical displacements due to this process follow the equation of a thin elastic plate floating over an inviscid fluid and it was solved using the finite-element method^44^. This model takes into account variable effective elastic thickness *T*_*e*_ following previous works that estimated this parameter over South America^45^. For the oceans and the western portion of the Andean chain, the *T*_*e*_ is 15 km, and for all other continental areas, the *T*_*e*_ is 70 km (see Fig. 4). The *T*_*e*_ along with the Young’s modulus *E*, the Poisson’s ratio ν and the density of each lithological layer are indicated in Table 1.

The surface processes include fluvial and marine transports of sediments. The fluvial transport of sediments occurs between cells separated by the steepest direction and follows the undercapacity model^46^ adapted to an irregular mesh^47^. In this formulation, the capacity to transport sediments from one cell to another is proportional to the topographic gradient and the water flux accumulated upstream. The lithologic information is given through the erosion length scale *L*, a parameter that controls the rock resistance to erosion^46^: regions with greater *L* are harder to erode. We set three different erosion length scale for the modeled region (see Table 1): (i) a high value for the shield areas *L*_*shields*_; (ii) an intermediate value for the basement *L*_*basement*_; and (iii) a low value for the sediments *L*_*sediments*_. The marine transport of sediments is simulated by a linear topographic diffusion with a constant diffusivity *k*_*m*_ and, for simplicity, the sea level was maintained constant at zero meters during all the simulations.

### Delimiting environments within amazonia

The Amazon rainforest is characterized by a densely and homogenous vegetation cover and exists at least since 50 Ma^48^. However, different edaphic conditions, that are a consequence of the drainage pattern and the regional geological settings among other features, can favor the development of different vegetation types within the rainforest, as well as geological processes can also diversify the paleoenvironments^49^.

Using our numerical results and taking into account the model limitations, mainly concerned to the spatial and temporal discretization, we were able to divide the landscape into regions that are susceptible to seasonally floods, the non-flooded areas, or terra firme, and the montane areas. In order to do that, we used an empirical method that takes into account the land topography and the water flux. As a constraint for our method to delimit the different environments within Amazonia, we used present estimates for the areal fraction of different environment in Western Amazonia^50^.

First, we delimited the terra firme areas of the model where the Voronoi cell satisfies the following adopted conditions:

(i) For each cell, the sum of the water flux of all neighboring cells should be lower than *Q*_*max*_ (see Table 2);

**Table 2:** Parameters used in the algorithm that delimits the different environments within Amazonia.

Parameter	Description	Value
*Q*_max_	Maximum water flux of neighboring cells to consider a cell as terra firme	5.0 × 10^11^ m^3^/year
*k*_0_	Empirical constant	1.25 × 10^−9^ year/m^2^
*h*_0_	Minimum altitude to consider a cell as Terra Firme	40 m
(ii) The topography of the cell must be greater than the value *h*_*min*_, which is calculated using the Eq. 1:

 

1 $${h}_{min}={h}_{0}+({Q}_{r}\,{k}_{0}),$$

where *h*_0_ is the minimum altitude for terra firme domains, *Q*_*r*_ is the water flux of the cell, and *k*_0_ is an empirical constant that is associated with the borderline of terra firme domain when comparing the water discharge and the altitude of a cell (see Table 2).

If one of the two previous conditions is not satisfied, the cell is considered susceptible to seasonally floods (yellow regions of Fig. 2).

The regions with topography higher than 1500 m are considered montane regions. To delineate the várzea domains (dark green lines on Fig. 2) we selected all the cells with headwaters in the Andean Cordillera. A similar method was used to trace the igapó regions (orange lines on Fig. 2), selecting the cells with headwaters in the shields. If a cell has headwaters both in the Andes and shields, we classified this cell as várzea. This procedure is used to differentiate the rivers originated in the Andes and the ones from the cratonic regions, representing different nutrient availability and provenance signature. Using different colors for the várzeas and the igapós we could illustrate the river path evolution during Andean orogeny.

## Supplementary information

Supplementary Information

Movie 1

Movie 2

Movie 3

Movie 4

Movie 5

Movie 6

## References

[1] Haffer J (1969). Speciation in amazonian forest birds. Science.

[2] Ribas CC, Aleixo A, Nogueira ACR, Miyaki CY, Cracraft J (2012). A palaeobiogeographic model for biotic diversification within Amazonia over the past three million years. Proc. Biol. Sci..

[3] Smith BT (2014). The drivers of tropical speciation. Nature.

[4] Naka LN, Brumfield RT (2018). The dual role of Amazonian rivers in the generation and maintenance of avian diversity. Science Advances.

[5] Silva SM (2019). A dynamic continental moisture gradient drove Amazonian bird diversification. Science Advances.

[6] Hoorn C (2010). Amazonia through time: Andean uplift, climate change, landscape evolution, and biodiversity. Science.

[7] Campbell KE, Frailey CD, Romero-Pittman L (2006). The Pan-Amazonian Ucayali Peneplain, late Neogene sedimentation in Amazonia, and the birth of the modern Amazon River system. Palaeogeogr. Palaeoclimatol. Palaeoecol..

[8] Latrubesse EM (2010). The late Miocene paleogeography of the Amazon Basin and the evolution of the Amazon River system. Earth Sci. Rev..

[9] Horbe AMC, Motta MB, de Almeida CM, Dantas EL, Vieira LC (2013). Provenance of Pliocene and recent sedimentary deposits in western Amazônia, Brazil: consequences for the paleodrainage of the Solimões-Amazonas River. Sediment. Geol..

[10] Nogueira ACR, Silveira R, Guimarães JTF (2013). Neogene-Quaternary sedimentary and paleovegetation history of the eastern Solimões Basin, central Amazon region. J. S. Am. Earth Sci..

[11] Rossetti DF (2015). Late Pleistocene OSL chronology in western Amazonia and implications for the transcontinental Amazon pathway. Sediment. Geol..

[12] Pupim FN (2019). Chronology of Terra Firme formation in Amazonian lowlands reveals a dynamic Quaternary landscape. Quat. Sci. Rev..

[13] Figueiredo J, Hoorn C, Van der Ven P, Soares E (2009). Late Miocene onset of the Amazon River and the Amazon deep-sea fan: evidence from the Foz do Amazonas Basin. Geology.

[14] Watts AB, Rodger M, Peirce C, Greenroyd CJ, Hobbs RW (2009). Seismic structure, gravity anomalies, and flexure of the Amazon continental margin, NE Brazil. J. Geophys. Res..

[15] Hoorn C (2017). The Amazon at sea: Onset and stages of the Amazon River from a marine record, with special reference to Neogene plant turnover in the drainage basin. Global and Planet. Change.

[16] Sacek V (2014). Drainage reversal of the Amazon River due to the coupling of surface and lithospheric processes. Earth Planet. Sc. Lett..

[17] Shephard GE, Müller RD, Liu L, Gurnis M (2010). Miocene drainage reversal of the Amazon River driven by plate–mantle interaction. Nat. Geosci..

[18] Eakin CM, Lithgow-Bertelloni C, Dávila FM (2014). Influence of Peruvian flat-subduction dynamics on the evolution of western Amazonia. Earth Planet. Sc. Lett..

[19] Flament N, Gurnis M, Müller RD, Bower DJ, Husson L (2015). Influence of subduction history on South American topography. Earth Planet. Sc. Lett..

[20] Roddaz, M. *et al*. In *Amazonia: Landscape and Species Evolution* (eds Hoorn, C. & Wesselingh, F.) 61–88 (Wiley-Blackwell, 2010).

[21] Paola C (2000). Quantitative models of sedimentary basin filling. Sedimentology.

[22] Wilkinson, M. J., Marshall, L. G., Lundberg, J. G. & Kreslavsky, M. H. In *Ama*zonia: Lan*ds*cape *and Species Evolution* (eds Hoorn, C. & Wesselingh, F.) 162–184 (Wiley-Blackwell, 2010).

[23] Crouch NMA, Capurucho JMG, Hackett SJ, Bates JM (2019). Evaluating the contribution of dispersal to community structure in Neotropical passerine birds. Ecography.

[24] Coronado ENH (2015). Phylogenetic diversity of Amazonian tree communities. Diversity Distrib..

[25] Dexter KG (2017). Dispersal assembly of rain forest tree communities across the Amazon basin. Proc. Natl. Acad. Sci. USA.

[26] Guedes TB (2018). Patterns, biases and prospects in the distribution and diversity of Neotropical snakes. Glob. Ecol. Biogeogr..

[27] Lynch Alfaro JW (2011). Explosive Pleistocene range expansion leads to widespread Amazonian sympatry between robust and gracile capuchin monkeys. J. Biogeogr..

[28] Schultz ED (2017). Systematics and biogeography of the Automolus infuscatus complex (Aves; Furnariidae): Cryptic diversity reveals western Amazonia as the origin of a transcontinental radiation. Mol. Phylogent. Evol..

[29] Ribas CC (2018). Biogeography and diversification of Rhegmatorhina (Aves: Thamnophilidae): Implications for the evolution of Amazonian landscapes during the Quaternary. J. Biogeogr..

[30] Tagliacollo VA, Roxo FF, Duke-Sylvester SM, Oliveira C, Albert JS (2015). Biogeographical signature of river capture events in Amazonian lowlands. J. Biogeogr..

[31] Dagosta FCP, De Pinna M (2017). Biogeography of Amazonian fishes: deconstructing river basins as biogeographic units. Neotrop. Ichthyol..

[32] Stokes MF, Goldberg SL, Perron JT (2018). Ongoing river capture in the Amazon. Geophys. Res. Lett..

[33] Quintero I, Jetz W (2018). Global elevational diversity and diversification of birds. Nature.

[34] Lima MGM (2017). Capuchin monkey biogeography: understanding Sapajus Pleistocene range expansion and the current sympatry between Cebus and Sapajus. J. Biogeogr..

[35] Ferreira M, Aleixo A, Ribas CC, Santos MPD (2017). Biogeography of the neotropical genus Malacoptila (Aves: Bucconidae): The influence of the Andean orogeny, Amazonian drainage evolution and palaeoclimate. J. Biogeogr..

[36] Byrne H (2018). Titi monkey biogeography: Parallel Pleistocene spread by Plecturocebus and Cheracebus into a post-Pebas Western Amazon. Zool. Scr..

[37] Thom G (2018). Phenotypic and genetic structure support gene flow generating gene tree discordances in an Amazonian floodplain endemic species. Syst. Biol..

[38] Lynch Alfaro JW, Izar P, Ferreira RG (2014). Capuchin monkey research priorities and urgent issues. Am. J. Primatol..

[39] Oberdorff T (2019). Unexpected fish diversity gradients in the Amazon basin. Science advances.

[40] Fjeldså J (1994). Geographical patterns for relict and young species of birds in Africa and South America and implications for conservation priorities. Biodivers. Conserv..

[41] Weir JT (2006). Divergent timing and patterns of species accumulation in lowland and highland neotropical birds. Evolution.

[42] Boubli JP (2015). Spatial and temporal patterns of diversification on the Amazon: A test of the riverine hypothesis for all diurnal primates of Rio Negro and Rio Branco in Brazil. Mol. Phylogent. Evol..

[43] Amante, C. & Eakins, B. W. ETOPO1 1 Arc-Minute Global Relief Model: Procedures, Data Sources and Analysis. NOAA Technical Memorandum NESDIS NGDC-24. National Geophysical Data Center, NOAA. 10.7289/V5C8276M (2009).

[44] Sacek V, Ussami N (2009). Reappraisal of the effective elastic thickness for the sub-Andes using 3-D finite element flexural modelling, gravity and geological constraints. Geophys. J. Int..

[45] Stewart J, Watts A (1997). Gravity anomalies and spatial variations of flexural rigidity at mountain ranges. J. Geophys. Res..

[46] Beaumont, C., Fullsack, P. & Hamilton, J. In: *Thrust Tectonics*. (eds: McClay, K. R.) 1–18 (Chapman and Hall, 1992).

[47] Braun J, Sambridge M (1997). Modelling landscape evolution on geological time scale: a new method based on irregular spatial discretization. Basin Res..

[48] Jaramillo, C. *et al*. In *Amazonia: Landscape and Species Evolution* (eds Hoorn, C. & Wesselingh, F.) 317–334 (Wiley-Blackwell, 2010).

[49] Irion, G., & Kalliola, R. In *Amazonia: Landscape and Species Evolution* (eds Hoorn, C. & Wesselingh, F.) 185–197 (Wiley-Blackwell, 2010).

[50] Toivonen T, Kalliola R, Mäki S (2007). The riverscape of Western Amazonia – a quantitative approach to the fluvial biogeography of the region. J Biogeogr..

