# Supplementary material for: Andean Tectonics and Mantle Dynamics as a Pervasive Influence on Amazonian Ecosystem
Source: Sci Rep. 2019 Nov 14;9:16879. doi: 10.1038/s41598-019-53465-y (PMC6856153; doi:10.1038/s41598-019-53465-y)
Supplement: Supplementary file 1 — Supplementary Information [file 41598_2019_53465_MOESM1_ESM.pdf]

Supplementary Information

Andean Tectonics and Mantle Dynamics as a Pervasive Influence on Amazonian Ecosystem

Tacio Cordeiro Bicudo*[1], Victor Sacek[1], Renato Paes de Almeida[2], John M. Bates[3], Camila Cherem Ribas[4].

[1]Instituto de Astronomia, Geofísica e Ciências Atmosféricas, Universidade de São Paulo, São Paulo, Brazil.

[2]Instituto de Geociências, Universidade de São Paulo, São Paulo, Brazil.

[3]Department of Zoology, The Field Museum, Chicago, U.S.A.

[4]Instituto Nacional de Pesquisas da Amazônia, Manaus, Brazil.

*Correspondence to: tacio.bicudo@iag.usp.br.

Supplementary Text

In Figures S1 and S2 we show the period of habitat stability since 4 Ma until the end of the simulations for the Model 1 and Model 3, respectively. Even though these numerical scenarios have different landscape evolutions previous to the onset of the transcontinental river system, after this moment the drainage configuration became very similar until the end of the simulation. This explains the similarity between the Figures 3, S1 and S2.

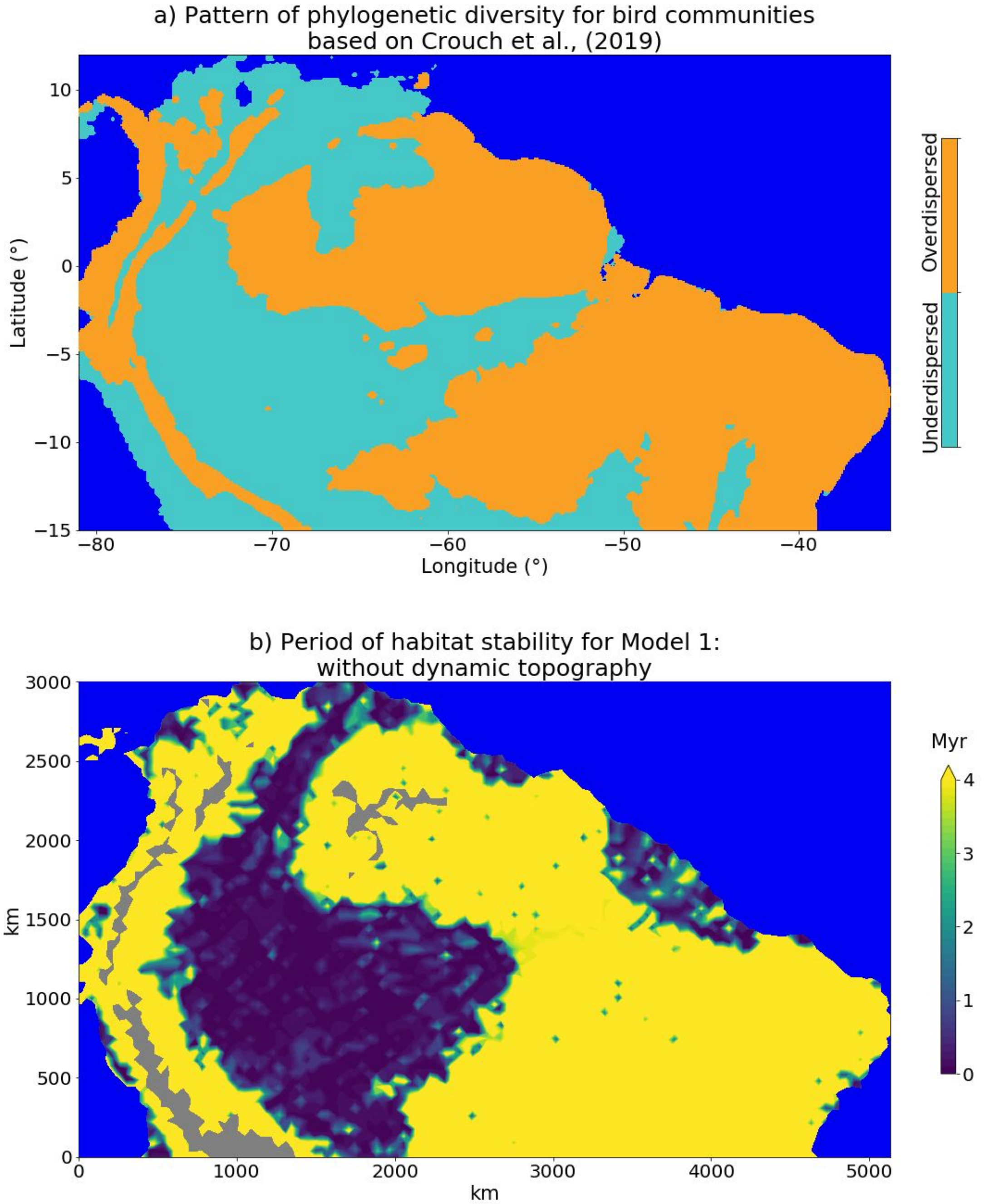

Supplementary Figure S1. **a)** Phylogenetic dispersion of bird communities in northern South America (adapted from Crouch et al.[1]). **b)** Period of uninterrupted habitat stability at the end of the simulation for Model 1, the model without the dynamic topography contribution. The grey areas are regions higher than 1500 m.

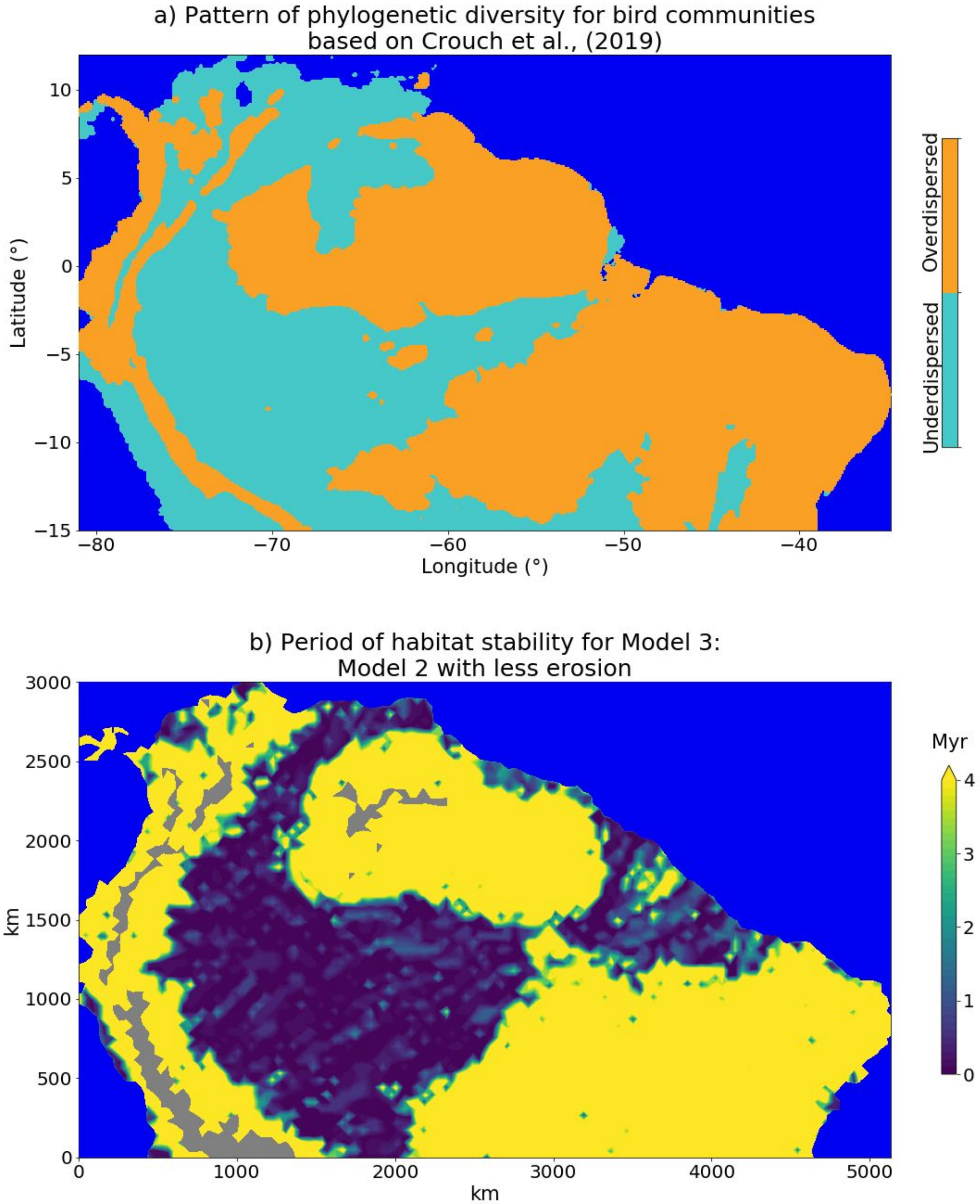

Supplementary Figure S2. a) Phylogenetic dispersion of bird communities in northern South America (adapted from Crouch et al.[1]). **b)** Period of uninterrupted habitat stability at the end of the simulation for Model 3, which has the same parameter of Model 2 but with a lower basement erosion rate. The grey areas are regions higher than 1500 m.

Geological constraints for the validation of the numerical scenarios

In this section we list the geological constraints that was used to validate the numerical scenarios.

01. The age of the onset of the transcontinental drainage system is estimated to be between 9 Ma and 10 Ma based on strong evidence from wells in the Foz do Amazonas Basin, i.e. lithology, sediment provenance and deposition rate[2,3], as well as palynological analysis from samples of the same wells[4]. These ages are also supported by the interpretations of seismic reflection profiles over the Amazon Fan in which is possible to observe an unconformity associated to a sea level fall at Late-Miocene and onset of the Andean derived sedimentation in the region[5]. Based on interpretations of sedimentary record of the intracratonic basins, in western and central Amazonia, some authors propose a much younger age for the onset of the transcontinental drainage, between Late Pliocene[6] and Pleistocene[7]. However, such young ages are difficult to sustain once the calculated amount of sediments deposited in the Amazon Fan[5] since the onset of the transcontinental drainage is too high to be deposited in a period shorter than 4 Myr, considering the actual observed suspended sediment discharge of the Amazon River near its mouth[8], and the evolution of the sedimentation rate deposition at the Amazon Fan, which monotonically increased since Late-Miocene[2]. In our numerical experiments, the transcontinental Amazon river is formed at 4.2, 14.2, and 10.4 Ma, for Models 1, 2 and 3, respectively. This timing is sensitive to the magnitude of dynamic topography, the initial elevation of the lowlands and the rate of exhumation of the Andean cordillera, but are compatible with the proposed ages based on the sedimentary record[2,4].
02. The sedimentary thickness estimated to be deposited since 35 Ma in the foredeep of the foreland basin system is approximately 3500 m[9], which is very close to the sedimentary thickness obtained in the foredeep of our numerical simulations. Eastward of the flexural forebulge the sedimentary thickness of the Miocene Solimões Formation can reach 1600 m[10],

which also is very close to our numerical results at the end of the simulation (see Figure S3).

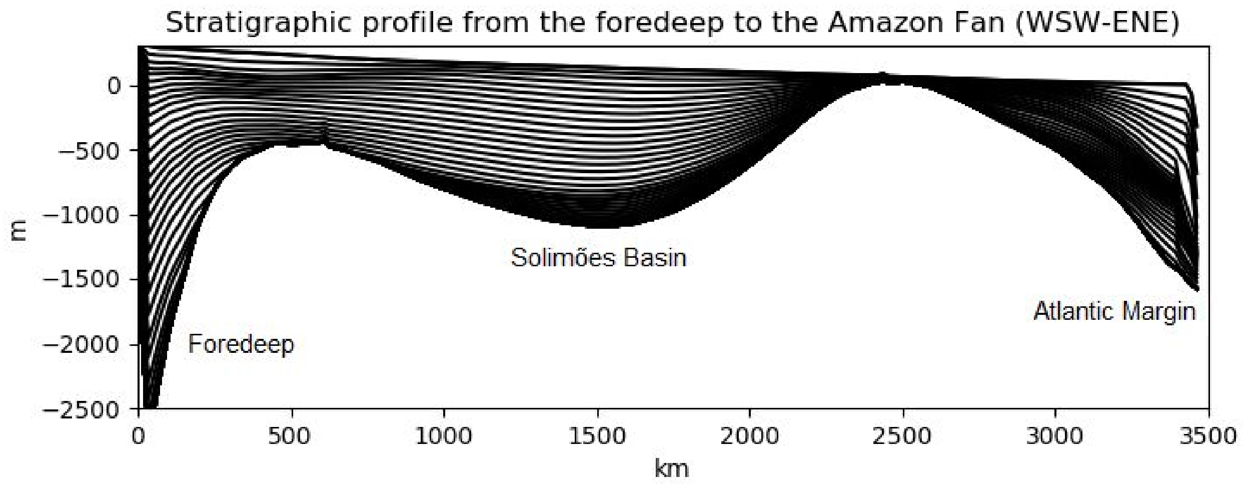

Supplementary Figure S3. Stratigraphic profile from the foredeep to the Amazon Fan for Model 2. The lines separate the sediments deposited in intervals of 1 Myr.

03. The age in which the tectonic activity of Northern Central Andes and Northern Andes achieved the present geographic dimensions of the actual Cordillera, which is proposed to be around 30 Ma[9,11-13], was used as the date for the onset of the numerical simulations. The paleoaltitude evolution calculated by Fiorella et al.[14] for the Bolivian Altiplano was used to compare with the mean altitude of the Central Andean Cordillera in the models (see Figure S4).

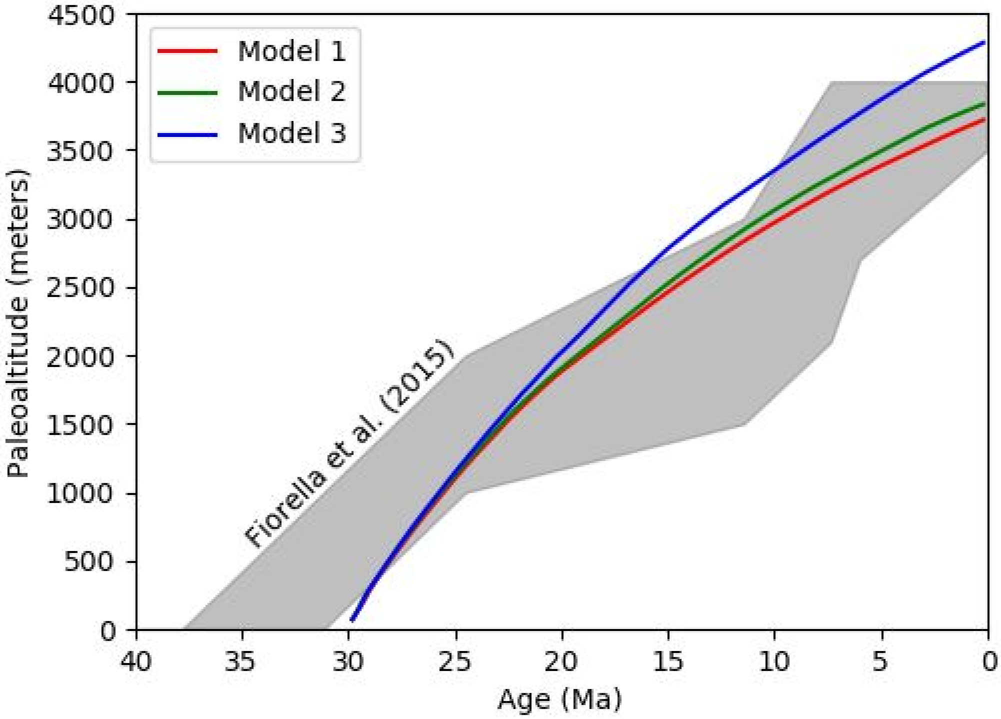

Supplementary Figure S4. Proposed paleolatitude evolution for the Bolivian altiplano (Fiorella et al.[14] based on analysis of cosmogenic isotope data (grey shaded domain), and mean altitude evolution of the Andean Cordillera between 15°S and 14°S for the Models 1, 2 and 3 (respectively the curves red, green and blue).

References

1. Crouch, N. M. A., Capurucho, J. M. G., Hackett, S. J. & Bates, J. M. Evaluating the contribution of dispersal to community structure in Neotropical passerine birds. *Ecography*, **42**, 390-399 (2019).

2. Figueiredo, J. P., Hoorn, C., Van der Ven, P. & Soares, E. Late Miocene onset of the Amazon River and the Amazon deep-sea fan: evidence from the Foz do Amazonas Basin. *Geology* **37**, 619–622 (2009).

3. Figueiredo, J. P., Hoorn, C., Van der Ven, P., & Soares, E. Late Miocene onset of the Amazon River and the Amazon deep-sea fan: Evidence from the Foz do Amazonas Basin: Reply. *Geology*, **38(7)**, e213-e213 (2010).

4. Hoorn, C., et al. The Amazon at sea: Onset and stages of the Amazon River from a marine record, with special reference to Neogene plant turnover in the drainage basin. *Global and Planet. Change* **153**, 51–65 (2017).

5. Watts, A. B., Rodger, M., Peirce, C., Greenroyd, C. J., & Hobbs, R. W. Seismic structure, gravity anomalies, and flexure of the Amazon continental margin, NE Brazil. *J. Geophys. Res.* **114**, 1-23 (2009).

6. Latrubesse, E.M., et al. The late Miocene paleogeography of the Amazon Basin and the evolution of the Amazon River system. *Earth Sci. Rev.* **99**, 99-124 (2010).

7. Rossetti, D.F., et al. Late Pleistocene OSL chronology in western Amazonia and implications for the transcontinental Amazon pathway. *Sediment. Geol.* **330**, 1-15 (2015).

8. Meade, R. H. in *Large Rivers: geomorphology and management* (eds Gupta, A.) 45-64 (John Wiley & Sons, 2008).

9. Roddaz, M., et al. in *Amazonia: Landscape and Species Evolution* (eds Hoorn, C. & Wesselingh, F.) 61-88 (Wiley-Blackwell, 2010).

10. Filho, J. R. W., Eiras, J. F., and Vaz, P. T. Bacia do Solimões. *Boletim de Geociências* da PETROBRAS, **15**(2), 217–225 (2007).

11. Parra, M., Mora, A., Sobel, E. R., Strecker, M. R., & González, R. Episodic orogenic front migration in the northern Andes: Constraints from low-temperature thermochronology in the Eastern Cordillera, Colombia. *Tectonics*, **28(4)**, 1-27 (2009).

12. Saylor, J. E., Stockli, D. F., Horton, B. K., Nie, J., & Mora, A. Discriminating rapid exhumation from syndepositional volcanism using detrital zircon double dating: Implications for the tectonic history of the Eastern Cordillera, *Colombia. Bulletin,* **124(5-6)**, 762-779 (2012).

13. Horton, B. K. Tectonic regimes of the central and southern Andes: Responses to variations in plate coupling during subduction. *Tectonics*, **37(2)**, 402-429 (2018).

14. Fiorella, R. P., Poulsen, C. J., Zolá, R. S. P., Jeffery, M. L., & Ehlers, T. A. Modern and long-term evaporation of central Andes surface waters suggests paleo archives underestimate Neogene elevations. *Earth and Planetary Science Letters*, **432**, 59-72 (2015).
